# Unexpected Vulnerability to High Temperature in the Mediterranean Alpine Shrub *Erysimum scoparium* (Brouss. ex Willd.) Wettst

**DOI:** 10.3390/plants10020379

**Published:** 2021-02-17

**Authors:** Águeda María González-Rodríguez, Eva María Pérez-Martín, Patricia Brito, Beatriz Fernández-Marín

**Affiliations:** Department of Botany, Ecology and Plant Physiology, University of La Laguna (ULL), 38200 San Cristóbal de La Laguna, Spain; evaperezmartin86@gmail.com (E.M.P.-M.); pbrito@ull.edu.es (P.B.); bfernanm@ull.edu.es (B.F.-M.)

**Keywords:** acclimation, alpine, cold, freezing, high mountain, hot, late spring frost, winter

## Abstract

Current understanding of the effects of extreme temperature on alpine evergreens is very limited for ecosystems under Mediterranean climate (characterised by a drought period in summer), despite being exceptionally biodiverse systems and highly vulnerable under a global change scenario. We thus assessed (i) seasonal change and (ii) effect of ontogeny (young vs. mature leaves) on thermal sensitivity of *Erysimum scoparium*, a keystone evergreen of Teide mountain (Canary Islands). Mature leaves were comparatively much more vulnerable to moderately high leaf-temperature (≥+40 and <+50 °C) than other alpine species. Lowest LT_50_ occurred in autumn (−9.0 ± 1.6 °C as estimated with *R_fd_*, and −12.9 ± 1.5 °C with *F_v_/F_m_*). Remarkably, young leaves showed stronger freezing tolerance than mature leaves in spring (LT_50_ −10.3 ± 2.1 °C vs. −5.6 ± 0.9 °C in mature leaves, as estimated with *R_fd_*). Our data support the use of *R_fd_* as a sensitive parameter to diagnose temperature-related damage in the leaves of mountain plants. On a global change scenario, *E. scoparium* appears as a well-prepared species for late-frost events, however rather vulnerable to moderately high temperatures.

## 1. Introduction

High mountain ecosystems are strongly determined by abiotic factors of the environment such as strong irradiance, wind, and extreme temperatures [1]. Temperature is in fact one of the most determinant factors in plant distribution globally [1]. Climatic extremes, such as late spring frost events, together with advanced vegetative periods, are crucial ecological and evolutionary drivers that can shift latitudinal and elevational distribution of temperate woody plants [2,3], while high temperature extremes and heat waves can lead to dramatic leaf damage and ultimately plant death, particularly when happening under water-limiting conditions [4,5,6,7]. Despite the overall trend towards decreasing air temperatures with elevation, plant leaves can be exposed to extreme high temperatures in alpine environments too [1,8]. In addition, a decoupling between leaf and air temperature (affected by wind, solar irradiance, transpiration, proximity to the soil, orientation, etc.) typically exacerbates leaf temperature extremes [8]. Seasonally, tolerance to extreme temperature varies along the year due to processes of acclimation/deacclimation (or hardening/dehardening) [9,10,11,12,13]. This fact is becoming of crucial relevance in the current context of global warming, since the spring phenology is advancing (due to overall temperature rise) faster than the date of the last spring frost.

In temperate ecosystems, tolerance to extreme temperatures in evergreen species differs amongst seasons, amongst individuals, and even amongst developmental stages or organs within a given individual, with young and reproduction-related organs generally being more sensitive [11,13,14]. With that respect, anatomical barriers for ice propagation towards leaf or flower buds in order to protect these sensitive organs are found in alpine woody plants [11,15]. Thus, the risk for frost-related damage has increased for deciduous and evergreen trees in the Alps over the last decades, particularly at high elevations (>800 m a.s.l.) [16]. Not only spring but also summer can represent a highly risky season for frost damage in alpine plants, due to their seasonal de-hardening, and this susceptibility can greatly vary with elevation within a single species [17].

Winter deacclimation is a crucial step that has however been much understudied [9]. Worryingly, mountain ecosystems are warming even faster than the global average [18,19] and the ecophysiological response of high mountain plants to heat stress is increasingly being addressed [10,20,21,22,23]. While irradiance seems to attenuate high temperature effects over their photosynthetic tissues by enhancing photoprotective and antioxidant responses, heat plus drought appears to be a catastrophic combination [22,23]. The response of high mountain evergreen shrubs to heat stress is comparatively much less understood than their response to low temperatures. In this context, an emphasis should be done in detailed seasonal monitoring of ecophysiological processes, such as photosynthesis, thermal regulation, and water use and transport. This would help us to accurately predict future changes in the distribution and fitness of species from high mountain ecosystems under Mediterranean climate, which are already being driven by the current global change scenario.

Our knowledge on the effects of extreme temperatures on alpine evergreens is much more limited for high mountains under Mediterranean climate than for high mountains under other climatic regimes. These are exceptional ecosystems because of their remarkable biodiversity and their unique combination of abiotic stresses that is mainly characterised by a drought period in summer, which strongly determines plants survival and adaptations [24]. An outstanding case study-site for these ecosystems is found in Teide Mountain, an up to 3718 m a.s.l. volcanic system in the subtropical Canary Island of Tenerife. The high mountain Mediterranean climate on Teide is characterised by very low precipitation (≤350 mm yearly). Air temperature can often drop below 0 °C in winter and rise above +25 in summer. This range is much more sharp above soil level (from −10 to +50 °C). An acute rise in temperature of +0.14 ± 0.07 °C per decade is occurring on Tenerife summit [25] and from the year 2000, 8 of the 10 hottest years in history have been recorded in the Teide National Park [26]. This ecosystem is preserved within the Teide National Park and accounts for 168 alpine species (32% of which are endemic) [27]. Scattered shrubs of a few taxa dominate the dry Supramediterranean belt [28], being *Spartocytisus supranubius*, *Pterocephalus lassiospermus,* and *Erysimum scoparium* remarkable keystone species. Some of these are experiencing significant changes in their distribution over the last couple of years [29]. Specifically, *Spartochytisus supranubius* is under regression being negatively affected by climate and alien herbivores, while *Pterocephalus lasiospermus* is under expansion being positively affected by warmer temperatures and from herbivore presence [29]. The Supramediterranean belt species are characterised by diverse leaf functional traits and photoprotective strategies [30,31] but very few studies have addressed the ecophysiological reasons behind these worrying alterations in their distribution. Overall, rise in minimum temperatures and drop in annual precipitation have been pinpointed as the most plausible factors, with additional pressure of herbivorism from invasive rodents [29,32,33,34,35]. In particular, severe drought stress plus overpressure of rodents seem to be co-responsible for a recession of *S. supranubius* [29,32], while nitrogen-related benefits from rabbit invasion, low palatability for this rodent, plus relatively high heat-tolerance seem to be co-responsible for a recent expansion of *P. lasiospermus* [29,33].

*E. scoparium* (Brouss. ex Willd.) Wettst is an evergreen shrub of the Brassicacea family, with linear and pubescent perennial leaves. The species is endemic to the Canary archipelago and naturally grows in the two islands with the highest elevations: La Palma and Tenerife where it grows in the subalpine areas within 1600 and 2200 m a.s.l [36]. *E. scoparium* is a representative species of the vulnerable Mediterranean high-mountain ecosystem of the Canary Islands. To date, susceptibility of *E. scoparium* to the current alterations of its natural habitat remains unknown. However, its capability to acclimate its photoprotection mechanisms across seasons is rather limited [31]. We thus aimed to evaluate how susceptible the photochemical performance of this species is to extreme temperatures since these are likely triggers of changes in its distribution over the coming years. Specifically, we addressed (i) the seasonal changes in thermal sensitivity and (ii) the effect of ontogeny (young vs. mature leaves) on the thermal sensitivity. We additionally compared two Chl*a*F-based parameters to estimate thermal sensitivity at leaf level (*F_v_/F_m_* and *R_fd_*) by using a Chl*a*F-imaging approach.

## 2. Results

### 2.1. Effects of Low-Temperature in E. scoparium Leaves

**Figure 1:**
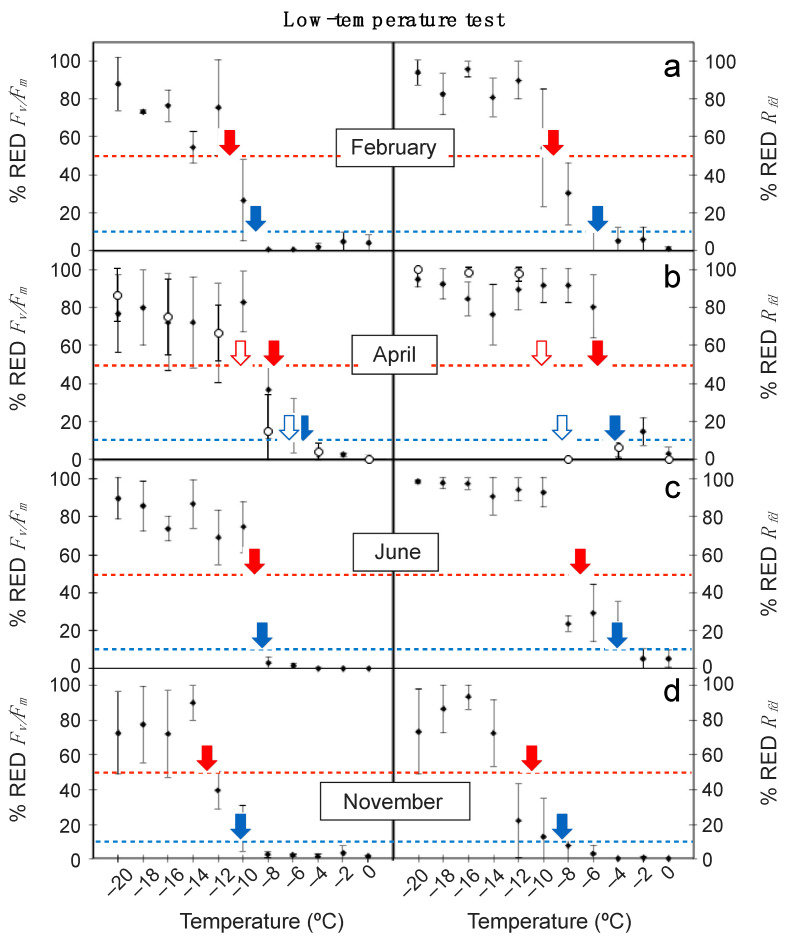
Effect of low temperature treatments on the reduction of F_v_/F_m_ (% RED *F_v_/F_m_*) and R_fd_ (% RED *R_fd_*) in mature leaves of *E. scoparium* along the year (close symbols) and in young leaves (open symbols) in April. Symbols are mean ± SD (*n* = 6, except for young leaves where *n* = 3). Red and blue arrows indicate the LT50 and the LT10, respectively, shown in Table 1.

The relationship between low temperature of the treatments and the obtained values for *% RED F_v_/F_m_* and *% RED R_fd_* is shown in Figure 1. Remarkably, the *% RED R_fd_* responded more sensitively to decreasing temperatures than *% RED F_v_/F_m_*. Thus, leaf damage (rise in *% RED*) occurred at higher temperatures for *% RED R_fd_* across all sampling seasons (Figure 1). Accordingly, the LT_10_ and LT_50_ values derived from the data shown in Figure 1 were also higher for *R_fd_* than for *F_v_/F_m_* (Table 1). Differences were particularly notable for the detection of incipient leaf damage (e.g., for LT_10_), as *R_fd_*-based results where several degrees higher (i.e., in February, LT_10_ was −9.1 ± 1.3 and −5.9 ± 1.6 °C for *F_v_/F_m_* and *R_fd_*_-_based data, respectively). Regardless *F_v_/F_m_* or *R_fd_*-based results were used, April was pinpointed as the most sensitive period to low temperature for adult leaves of *E. scoparium* (Figure 1). The lowest LT_10_ and LT_50_ of the year were obtained in this month, and a decrease in *R_fd_* occurred already at temperatures as high as −4.1 ± 1.3 °C (Table 1). By contrast, November was the less sensitive period (Table 1, Figure 1). Unexpectedly, mature leaves were more sensitive to low temperature than young ones for the tested season: April. Differences were already substantial in the plots of temperature against *% RED R_fd_*. As an example, an abrupt reduction in *R_fd_* occurred at temperature <−4 °C in mature leaves, and <−8 °C in young ones (Figure 1). This led to important differences in the LT_50_, particularly outstanding when estimated from *R_fd_*: LT_50_ was −5.6 ± 0.9 °C in the mature, and −10.3 ± 2.1 °C in the young leaves (Table 1).

**Figure 2:**
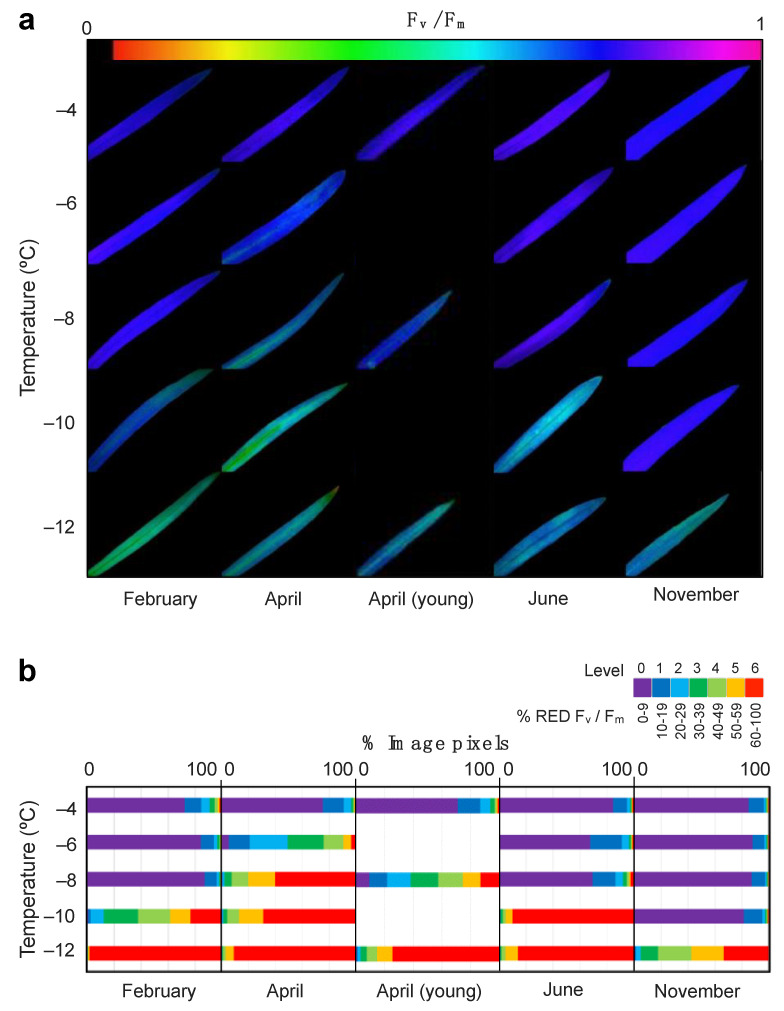
Effect of low temperature treatment on *E. scoparium* leaves as obtained from chlorophyll fluorescence imaging analysis. (**a**) Representative images of chlorophyll fluorescence showing F_v_/F_m_ values of whole leaves after selected temperature treatments. (**b**) Summary of the quantitative analyses of F_v_/F_m_ images. The % of reduction in the F_v_/F_m_ was classified in seven stress levels as shown in the legend (violet: Low to non-reduction, red: Dramatic reduction). The length of the horizontal colour bars is proportional to the number of image pixels of each stress-level.

As revealed by Chl*a*F imaging, low temperature effect was heterogeneous across the leaf blade (Figure 2a). The semi-quantitative analyses of the Chl*a*F images revealed the coverage of the damaged area (Figure 2b) highlighting the differences across sampling months. Hence, the highest tolerance to freezing occurred in November. This was the only month without an apparent leaf rise in *% RED F_v_/F_m_* at temperatures as low as −10 °C (Figure 2b).

### 2.2. Effects of High-Temperature in E. scoparium Leaves

**Figure 3:**
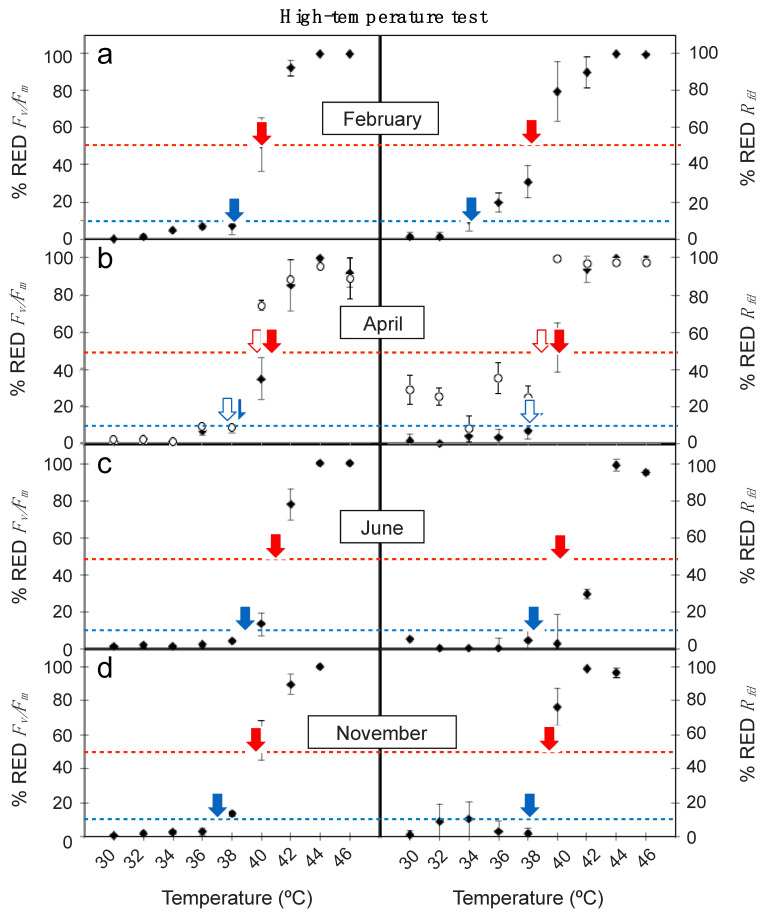
Effect of high temperature treatments in the reduction of *F_v_/F_m_* (% RED *F_v_/F_m_*) and R_fd_ (% RED *R_fd_*) in mature leaves of *E. scoparium* along the year (close symbols) and in young leaves (open symbols) in April. Symbols are mean ± SD (*n* = 6, except for young leaves where *n* = 3). Red and blue arrows indicate the LT_50_ and the LT_10_, respectively, shown in Table 2.

Obtained values for *% RED F_v/_F_m_* and *%* RED *R_fd_* plotted against high temperature treatments are shown in Figure 3. Again, the *%* RED *R_fd_* responded more sensitively to increasing temperatures than *% RED F_v/_F_m_*. Thus, for a given temperature (i.e., 40 °C) signs of leaf damage (rise in % RED) were more pronounced for *% RED R_fd_* across all sampling seasons (Figure 3). Accordingly, the LT_10_ and LT_50_ values derived from the relationships were also lower for *R_fd_* than for *F_v_/F_m_* (Table 2). Differences were particularly notable for the detection of incipient leaf damage (e.g., for LT_10_), as *R_fd_*-based results where up to several degrees lower (i.e., in February, LT_10_ was 34.2 ± 1.2 and 38.1 ± 1.1 °C for *R_fd_* and *F_v_/F_m_*-based data, respectively). Regardless *F_v/_F_m_* or *R_fd_*-based results were used, June was pinpointed as the less sensitive period to high temperature for adult leaves of *E. scoparium* (Figure 3). The lowest LT_10_ and LT_50_ of the year were obtained in this month, and a decrease in *R_fd_* occurred only at temperatures >40 °C (Figure 3, Table 2). By contrast, February (closely followed by November) was the more sensitive period (Figure 3, Table 2). Contrasting with the obtained results for low-temperature treatments, mature young leaves were more sensitive to high temperature than mature ones in April (Figure 3, Table 2). Differences were already substantial in *% RED F_v_/F_m_*: i.e., the treatment of +40 °C induced a decrease of 35% in mature leaves but of 75% in the young leaves. Even more drastic were the results obtained with *% RED R_fd_*: a decrease of 50% took place in mature leaves but of 100% in the young leaves (Figure 3).

**Figure 4:**
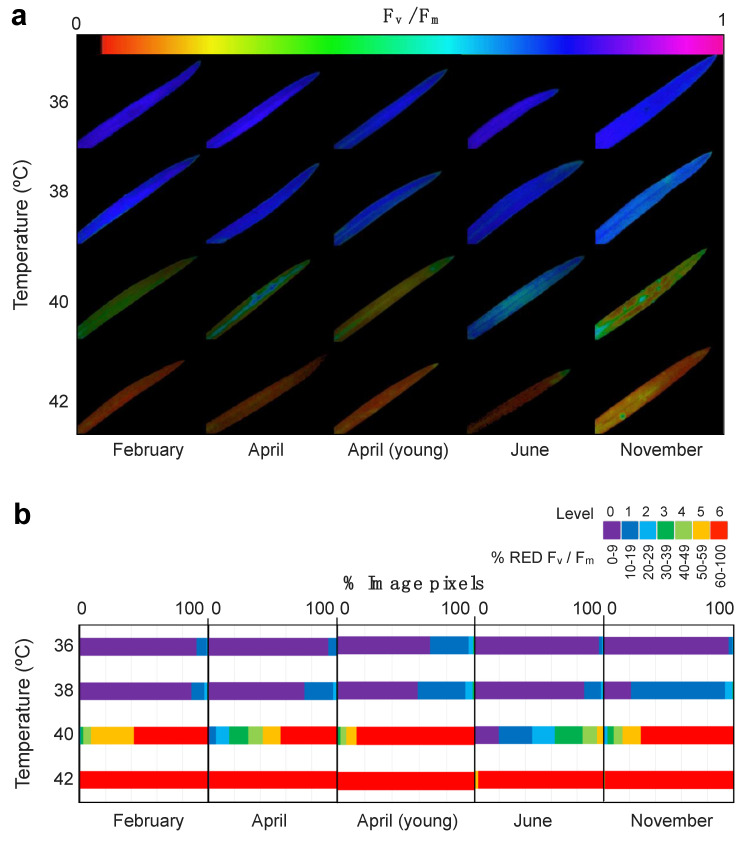
Effect of high temperature treatment on *E. scoparium* leaves as obtained from chlorophyll fluorescence imaging analysis. (**a**) Representative images of chlorophyll fluorescence showing F_v_/F_m_ values of whole leaves after selected temperature treatments. (**b**) Summary of the quantitative analyses of F_v_/F_m_ images. The % of reduction in the F_v_/F_m_ was classified in seven stress levels as shown in the legend (violet: Low to non-reduction, red: Dramatic reduction). The length of the horizontal colour bars is proportional to the number of image pixels of each stress-level.

The spatial pattern of leaf damage due to high temperature as revealed by Chl*a*F imaging, is showing Figure 4. The semi-quantitative analyses of the Chl*a*F images revealed the coverage of the damaged area (Figure 4b) highlighting the differences across sampling months. Hence, the highest tolerance to high temperature did happen in June, when even at +40 °C less than half of the leaf area showed strong *% RED F_v_/F_m_* (i.e., Level ≥ 5 of damage). November was the most sensitive month with more than 60% of the leaf area showing the highest level of damage (level 6) after the +40 °C treatment (Figure 4b). Even more clearly was June as the most tolerant month to elevated temperatures with no apparent rise in *% RED F_v_/F_m_* at +40 °C.

## 3. Discussion

### 3.1. Low Temperature and Mature Leaves of E. scoparium

Mature leaves of *E. scoparium* showed its highest tolerance to freezing in autumn (November) and its lowest, in spring (April) (see the non-overlapped confident intervals, in Table 1 and Table 2). The results obtained with *F_v_/F_m_* analyses indicate that *E. scoparium* leaves are more tolerant to freezing in early summer (LT_50_ June = −9.3 °C) than in spring (LT_50_ April = −5.5 °C). This contrasts with data obtained in temperate mountain regions such as the Alps, where June is frequently amongst the most sensitive months to frost damage [17,37]. Being a Mediterranean species, drought and high-irradiance acclimation of *E. scoparium* could be plausible processes related to its enhancement of freezing tolerance towards summertime. In that sense, freezing resistance can be enhanced by drought in plants from arid mountains species in the Andes [38] and physiological processes, particularly related to photoprotection, are part of the cross-tolerance of leaves to desiccation and freezing in European mountain plants within the Mediterranean Basin [39,40,41]. In agreement with this, the topsoil (e.g., uppermost 10 cm) keep generally completely dry from June along the summer in the habitat of *E. scoparium* [42]. In addition, this species shows a robust photoprotection system that is maintained relatively constant across seasons [31]. The results obtained with *R_fd_* analyses showed the same trend but with smallest differences between early summer (LT_50_ June = −6.9 °C) and spring (LT_50_ April = −5.6 °C).

**Figure 5:**
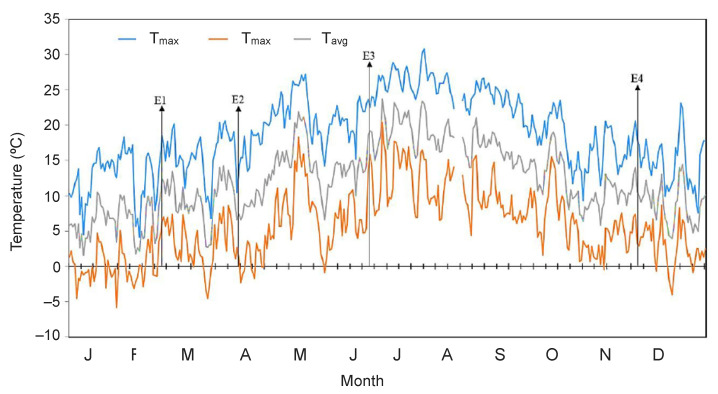
Daily temperature at Los Roques (2070 m a.s.l within the Teide National Park) in Tenerife, Canary Islands during the year of study. Daily mean and absolute minimum and maximum air temperature values are shown. Arrows highlight the sampling days for each experiment: E1, 23th of February (winter); E2, 8th of April (spring); E3 22nd of June (early summer); E4, 23rd of November (autumn).

An unexpected result obtained for *E. scoparium* was then the high frost sensitivity of its mature leaves in April (Table 1), mostly considering subzero temperature events can happen during this month (Figure 5). In this regard, an advance in spring phenology (due to overall temperature rise) faster than the date of the last spring frost, has been argued as a main factor rising the risk for frost-related damage of trees in the Alps at elevations >800 m a.s.l. [16]. This observation in the Alps is in agreement with the still understudied process of winter-deacclimation in evergreens [9] and with the generally accepted idea of high sensitivity to frost of young (e.g., new-sprouting leaves) when compared to mature tissues of a same species or individuals. Nevertheless, as discussed below in Section 3.3, this is not exactly the case of *E. scoparium*, since its new emerging leaves show higher tolerance to low temperature in the same month (April) (Figure 1 and Figure 2, Table 1).

### 3.2. High Temperature and Mature Leaves of E. scoparium

*E. scoparium* showed highest tolerance to heat in April-June. High irradiance enhances heat tolerance [20,22,23], which could at least partially explain the results even if our experiments were conducted in darkness, i.e., irradiance strongly rises from march to june in the natural environment were the leaves were collected. Additionally, heat-tests performed in darkness with alpine plants, also showed *F_v_/F_m_*-based LT_50_ higher ≥+45 °C [23]. Extreme high temperature is, together with drought, one of the most affecting factors to Mediterranean plants [43]. Thus, even when naturally acclimated to a significant variation of temperatures seasonally, severe rises in temperature (such as heat waves) can affect Mediterranean woody plants reversibly or irreversibly [6,44]

*E. scoparium* is comparatively much sensitive to high temperatures than previously tested alpine species. The maximum temperatures for LT_50_ obtained for *E. scoparium* were around +40 °C which is surprisingly lower temperature than that obtained for alpine shrubs at higher latitudes (i.e., in ≈ +50 °C in the Alps) [45]. By compiling experiments of different research groups performed with alpine species from the 1960s, the same authors [10] have emphasised that 42 °C is the lowest heat killing temperature of the most susceptible alpine plant species. The susceptibility of *E. scoparium* leaves to high temperature is particularly unexpected considering two aspects. First, plant leaves can rise their temperature several degrees over the air temperature due to high irradiance [8,10]. Second, over the last two decades (2000–2020), 8 out of the 10 hottest years ever seen have been registered in the natural habitat of this endemic species (Teide National Park) (Martin-Esquivel and Pérez-González, 2019). In this scenario, this ecosystem would very likely lose diversity, since only the more heat tolerant species would survive [33].

### 3.3. Young Leaves of E. scoparium Are Remarkably Freezing-Tolerant but Unexpectedly Vulnerable to High Temperatures

Most unexpected results in our work were obtained with young leaves of *E. scoparium* measured in spring (April). Vegetative growth periods are spring and autumn in Mediterranean habitats, and this concerns to the species growing at Teide high mountain ecosystem too [32,34]. Interestingly, new leaves of *E. scoparium* sprout in February, which can be the coldest month in the year (Figure 5). This can be the reason behind their remarkable freezing-tolerance when compared to young leaves of other alpine species. For the latter, it has been typically described that young leaves are freezing sensitive, suffering frost damage at temperatures close to those of ice formation [11], while LT_50_ of *E. scoparium* was ≤−10 °C (Table 1). Interestingly, most alpine species sprout comparatively later, typically during late spring-early summer (e.g., June), concomitant with the starting of the growing season. New-sprouted leaves of *E. scoparium,* though, encounter a very different environmental context: e.g., the end of winter (Figure 5). Their surprising high tolerance to freezing, on the one hand, highlights their acclimation and, on the other hand, reveals the need for further ecophysiological studies to Mediterranean mountain ecosystems.

Contrasting with their remarkable tolerance to freezing, young leaves of *E. scoparium* were unexpectedly sensitive to moderately high temperatures with LT_50_ ≤ +40 °C (Table 2). These values indicates a risk of damage even when maximum air temperature in April is around +20 °C, because the elevated irradiation at this time of the year can induce a strong decoupling between leaf and air temperatures. As illustrative examples, the canopy temperature of antarctic mosses can exceed in 15 °C the temperature of the air at irradiances ≥ 800 μmol m^−2^ s^−1^ [46]; while leaf temperatures ≥ +50 °C have been measured in alpine plants during summer-months [10]. Both cases are comparable to April in Teide, in terms of irradiance, considering its low latitude, and that irradiance can easily overpass 1000 μmol m^−2^ s^−1^ during sunny days [31,47]. Furthermore, it is noteworthy that photosynthesis (e.g., net carbon assimilation) is limited by heat at around 3 °C lower than visible symptoms of injure appear in the leaves [48]. All things considered, young leaves from *E. scoparium* are unexpectedly susceptible to moderately high temperature.

### 3.4. R_fd_ and ChlaF Imaging: Useful Tools to Diagnose Temperature-Induced Leaf-Damage

Leaf damage induced by temperature stress can be qualitatively and quantitatively estimated through different approaches. Most widely used methods in high mountain studies include direct assessment of visual injures, electrolyte leakage, viability evaluation through the tetrazolium test, and chlorophyll *a* fluorescence (Chl*a*F) [5,10,13,33]. Among them, Chl*a*F-imaging seems a promising tool, because it enables the characterization of either acclimation or damage against temperature treatments at leaf and at plant levels [49,50]. Importantly, this method provides information on the spatio-temporal pattern of temperature effects on leaf photochemistry [51,52]. While the maximum photochemical efficiency of photosystem II (*F_v_/F_m_*) has traditionally been used in the early-diagnosis of temperature-derived damage in photosynthetic tissues of mountain plants [22,53], a recent study has pinpointed the Chl*a*F decrease ratio, as a more sensitive parameter [33]. This so-called vitality index (*R_fd_*), was introduced in the 1980s [54], relates to the leaf photochemical capacity under continuous irradiance, and strongly correlates with carbon assimilation [55]. To date, relatively few studies have taken advantage of the Chl*a*F-imaging analyses to provide spatio-temporal patterns in alpine leaf damage under temperature stress [51,52], and none of them has focused on the *R_fd_* parameter.

Amongst the different parameters obtained from Chl*a*F measurements that provide valuable information on leaf photochemical fitness, *F_v_/F_m_* is one of the most widely used [5,10,13,33]. This is an easy and fast measurement since only pre-acclimation of the photosynthetic tissue to darkness is needed before a short (typically <1 s) saturating light is applied with the fluorometer. Nevertheless, slightly longer measurements that require a controlled illumination of the sample (e.g., 5 min) provide even more accurate parameters such as the *R_fd_*, as already highlighted by Lichtenthaler [54] and more recently foster by several other authors [30,33,56]. Our results with *E. scoparium* further support these previous studies (Table 1 and Table 2). Thus, *R_fd_* could be used as a pre-emptive estimator of leaf damage induced by temperature.

## 4. Materials and Methods

### 4.1. Field Site and Experimental Design

Study site was located in Los Roques at 2070 m a.s.l, within the Teide National Park (TNP) in Tenerife, Canary Islands (28°18′ N, 16°34′ W). Three to four adult plants of the case study species *Erysimum scoparium* (Brouss. ex Willd.) Wettst. were randomly selected from three different plots in the study site. All plants were similar in size and were grown under comparable conditions in a S-SW exposed area with a slight slope. A seasonal sampling was conducted with four field campaigns the 23th February (winter), 8th of April (middle spring), 22nd of June (early summer), and 23rd of November (autumn) in 2015 (Figure 5). The air temperature depicted in Figure 5 was obtained from the AEMET Meteorological Station (code C406G) located at approximately 300 m from the study site. Only fully developed healthy leaves were collected for measurements, from the third to fifth node from the apex of the stem. Fully expanded young leaves (approximately 2-month old) were additionally collected in April and compared to the adult leaves. All leaves were 6–8 mm width and 5–6 m long. Leaves were preserved at 100% relative humidity at 18 °C in the dark and immediately brought to the lab. After 24 h of dark incubation, the initial maximal photochemical efficiency of PSII (*F_v_/F_m_*) was measured, and only samples showing values above 0.75 were used for the temperature treatments, as detailed below.

### 4.2. Temperature-Tolerance Treatments

The procedure of a standard temperature-tolerance test was followed [5,33]. Six mature and three young selected leaves from different plants were randomly selected for each temperature treatment, assuring that all leaves came from a different plant within each analyses. During the treatments the samples were kept inside a plastic bag. The bag contained a wet tissue to prevent sample dehydration. A digital thermocouple (Thandar TH 302, TTi, Thurlby Thandar Instruments, Cambridgeshire, UK) was attached to one of the leaves and the temperature monitored along the treatment. Samples were then introduced into a thermostatic water bath (Hetofrig CB11E, Heto, Birkerød, Denmark). Target temperature was kept for 30 min. Each sample set was subjected to one of the following target temperatures: High: +30, +32, +34, +36, +38, +40, +42, +44, +46 °C; or low temperatures: 0, −2, −4, −6, −8, −10, −12, −14, −16, −18, −20 °C. Thus, 120 adult and 60 young leaves were measured each season (420 leaves, in the whole study). All temperature treatments were performed in darkness. The temperature-damage was estimated through chlorophyll fluorescence as described below.

### 4.3. Chlorophyll Fluorescence Analyses

An Imaging-PAM system (Mini blue version, Walz GmbH, Effeltrich, Germany) was used for chlorophyll fluorescence (Chl*a*F) measurements. This enabled an in deep analysis of the changes induced by temperature in the photochemical performance, but also of their spatial distribution within the leaves. Different parameters were obtained as follows just before and immediately after each temperature treatment. First, F_0_ was measured in the dark-adapted sample. Second, a saturating light pulse (3000 μmol m^−2^ s^−1^) was applied to determine F_m_. After that, a saturating and continuous actinic light (1175 μmol m^−2^ s^−1^) was switched on and the decrease of the fluorescence was recorded for 5 min until a steady state (F_s_) was reached. From these light curves, the F_0_, F_m_, and F_s_ were obtained to calculate: *F_v_/F_m_* as (F_m_–F_0_)/F_m_, and *R_fd_* as (F_m_–F_s_)/F_s_) following [55]. The whole area of the leaf was considered and the final *F_v_/F_m_* or *R_fd_* (after temperature treatment) was expressed as % of decrease in comparison to the initial value, for each leaf individually. We obtained in that way the parameters thereafter referred as *% RED F_v/_F_m_* and *% RED R_fd_.* From these values we thereafter estimated the temperature that induced a 10% of decrease in the initial *F_v_/F_m_* and *R_fd_* values, and the temperature that induced a 50% of decrease (LT_10_ and LT_50_) according to [13]. The analyses of Chl*a*F images enabled us to obtain further information, e.g., which proportion of the leaf (measured as % of pixels) was affected, and to which extent (by comparing *F_v_/F_m_* value of each pixel after temperature treatment, with the average of all pixels from the leaf before the treatment). For this purpose, the number of pixels and their average F_v_/F_m_ value, within each leaf, was obtained at t_0_. Thereafter the *F_v_/F_m_* values obtained after the temperature treatments were expressed, for each pixel, as % of reduction compared to the initial average value (*% RED F_v_/F_m_*). Next, we classified these *% RED F_v_/F_m_* values in 7 levels: Level 0 to 6 corresponding to 0–9%, 10–19%, 20–29%, 30–39%, 40–49%, 50–59%, and 60–100% *RED F_v_/F_m_*, respectively. Finally, the % of pixels with each level was also quantified per image individually, and then averaged per treatment.

### 4.4. Statistical Analyses

The LT_50_, and the LT_10_ were estimated from a linear regression fitted to the central (linear) part of the sigmoid relationship between the damaging temperature and the percentage of damage. A minimum of least three temperatures within this linear central part were used [57,58]. We then obtained the regression models with StatGraphics (Centurion XVI.I, StatPoint Technologies, Inc., Rockville, MD, USA) after normality and homoscedastic hypothesis had been tested. In addition to the mean values of LT_50_ and LT_10_, the upper and lower limits of the intervals were estimated with a confident interval of 95% (α = 0.05) (Table 1 and Table 2) and the intercepts and slopes of the regression models were compared by F-test.

## 5. Conclusions

The representative species of the vulnerable Mediterranean high-mountain ecosystem of the Canary Islands *Erysimum scoparium*, is sensitive to leaf-temperature above +40 °C. Its thermal sensitivity to high temperature is rather constant across seasons. This species is, by contrast, rather tolerant to sub-zero leaf-temperature, particularly at the end of autumn (November). Remarkably, young leaves showed stronger freezing tolerance than mature leaves but comparable sensitivity to high temperature. Finally, our data support the use of *R_fd_* as a sensitive parameter to diagnose temperature-related damage in the leaves of mountain plants. Further ecophysiological studies on mountain ecosystems under Mediterranean climate, which are so far rather scarce, are needed.

## Figures and Tables

**Table 1:** LT_10_ and LT_50_ estimated from low temperature treatments from the maximal photochemical efficiency of PSII (*F_v_/F_m_*) and the *R_fd_* data shown in Figure 1 and obtained from the regression model. Values are mean ± confidence interval. Significance (*p*-value) and fit (R^2^) to the regression are shown.

	LT	(°C) *F_v_/F_m_*	*p*-Value	R^2^	(°C) *R_fd_*	*p*-Value	R^2^
February	**LT_10_**	−9.1 ± 1.3	0.000	0.72	−5.9 ± 1.6	0.000	0.74
	**LT_50_**	−10.7 ± 1.1			−8.7 ± 1.3		
April	**LT_10_**	−5.5 ± 1.3	0.000	0.75	−4.1 ± 1.3	0.000	0.81
	**LT_50_**	−7.8 ± 1.2			−5.6 ± 0.9		
April (young leaves)	**LT_10_**	−6.2 ± 3.5	0.003	0.75	−8.6 ± 2.8	0.010	0.92
	**LT_50_**	−10.1 ± 3.6			−10.3 ± 2.1		
June	**LT_10_**	−8.3 ± 0.5	0.000	0.94	−4.1 ± 1.8	0.000	0.67
	**LT_50_**	−9.3 ± 0.4			−6.9 ± 1.5		
November	**LT_10_**	−10.2 ± 1.9	0.000	0.61	−8.5 ± 3.2	0.000	0.67
	**LT_50_**	−12.9 ± 1.5			−11.1 ± 1.6		

**Table 2:** LT_10_ and LT_50_ estimated from high temperature treatments from the maximal photochemical efficiency of PSII (*F_v_/F_m_*) and the *R_fd_* data shown in Figure 3 and obtained from the regression model. Values are mean ± confidence interval. Significance (*p*-value) and fit (R^2^) to the regression are shown.

	LT	(°C) *F_v_/F_m_*	*p*-Value	R^2^	(°C) *R_fd_*	*p*-Value	R^2^
February	**LT_10_**	38.1 ± 1.1	0.000	0.90	34.2 ± 1.2	0.000	0.91
	**LT_50_**	40.3 ±0.7			38.2 ± 0.8		
April	**LT_10_**	38.3 ± 0.9	0.000	0.91	38.3 ± 0.7	0.000	0.93
	**LT_50_**	40.6 ± 0.6			40.0 ± 0.5		
April (young leaves)	**LT_10_**	37.9 ± 1.7	0.000	0.88	38.2 ± 0.07	0.000	0.99
	**LT_50_**	39.7 ± 1.1			39.0 ± 0.1		
June	**LT_10_**	39.0 ± 0.8	0.000	0.91	38.3 ± 0.8	0.000	0.66
	**LT_50_**	41.1 ± 0.6			40.1 ± 0.5		
November	**LT_10_**	37.1 ± 0.8	0.000	0.94	38.1 ± 1.1	0.000	0.87
	**LT_50_**	39.8 ± 0.6			39.6 ± 0.7		

## Data Availability

Data from this study are available from the corresponding author A.M.G.-R. upon request.
